# Case Report: Successful pregnancy after fertility-sparing surgery for ovarian clear cell carcinoma

**DOI:** 10.3389/fonc.2026.1735073

**Published:** 2026-02-11

**Authors:** Shubo Wang, Qian Li, Bingxiu Zhou, Fuxin Zhou

**Affiliations:** 1 Department of Gynecology, The Affiliated Weihai Second Municipal Hospital of Qingdao University, Weihai, China; 2 Department of General and Visceral Surgery, Faculty of Medicine, Medical Center – University of Freiburg, Freiburg, Germany

**Keywords:** adjuvant chemotherapy, fertility-sparing surgery, *in vitro* fertilization, ovarian cell carcinoma, successful pregnancy

## Abstract

Ovarian clear cell carcinoma (OCCC) is a rare histologic subtype of epithelial ovarian cancer, often associated with chemoresistance and poor prognosis. Fertility preservation in patients with OCCC remains controversial because of its aggressive biological behavior and limited evidence supporting conservative management. We report a case of a 33-year-old woman with stage IA OCCC who underwent fertility-sparing staging surgery followed by six cycles of adjuvant chemotherapy with liposomal paclitaxel and carboplatin. Gonadotropin-releasing hormone agonist was administered concurrently to protect ovarian function. The follow-up duration was 36 months, during which the patient remained disease-free and achieved a successful full-term pregnancy after *in vitro* fertilization and embryo transfer. The pregnancy resulted in a full-term delivery, and the patient gave birth by cesarean section without complications. This case demonstrates that fertility-sparing surgery combined with adjuvant chemotherapy may be a feasible treatment option in carefully selected young patients with early-stage OCCC. Individualized management, comprehensive counseling, and close surveillance are essential to ensure both oncologic safety and favorable reproductive outcomes.

## 1 Introduction

Epithelial ovarian carcinomas can be grouped into five main histologic subtypes by different histopathological features and molecular pathogenesis, namely, high-grade serous, low-grade serous, endometrioid, ovarian clear cell carcinoma (OCCC), and mucinous carcinoma (1). OCCC is the second most common histologic subtype of epithelial ovarian cancer (EOC) after the serous type and typically occurs in younger women at an early stage (2–4). It accounts for approximately 10% of all ovarian carcinomas, with reported incidence rates ranging from 5% to 25% depending on geographic, ethnic, and racial factors (1, 5, 6). In recent years, the incidence of OCCC has increased in several countries, with the highest rates observed among Asian women.

Given the younger age at diagnosis and early-stage presentation, fertility preservation has become an important consideration for selected patients with OCCC. In a large cohort of young patients with stage I OCCC, fertility-sparing surgery (FSS) was not associated with worse survival (7). However, OCCC has a high risk of recurrence, largely due to its relative resistance to platinum-based chemotherapy (8, 9). Consequently, fertility preservation in patients with OCCC remains controversial owing to its aggressive biological behavior and limited evidence supporting conservative management (10).

Herein, we report a young patient with OCCC who underwent a fertility-sparing operation, followed by six courses of combination chemotherapy with liposomal paclitaxel and carboplatin, and remained disease-free during follow-up and achieved a successful full-term pregnancy after *in vitro* fertilization and embryo transfer.

## 2 Case presentation

**Figure 1:**
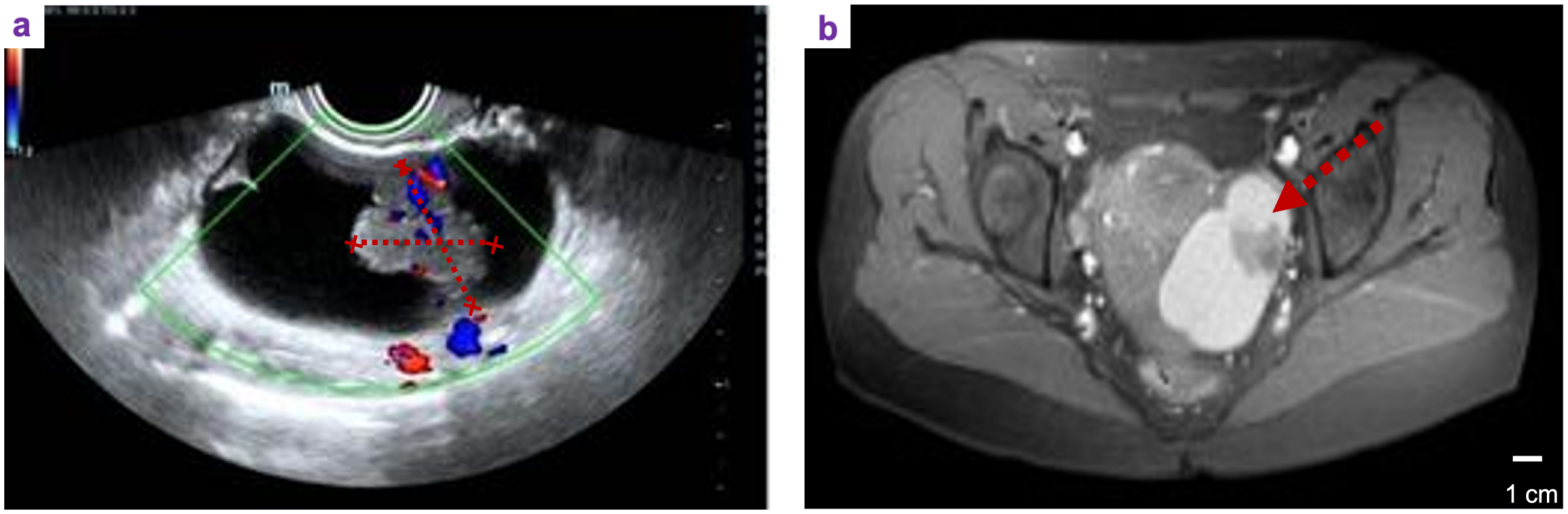
Preoperative imaging findings. **(a)** Transvaginal ultrasonography demonstrating a mixed cystic–solid mass in the left adnexal region with internal septations and increased vascularity on color Doppler imaging (arrow). **(b)** Axial pelvic magnetic resonance imaging (MRI) showing a cystic–solid lesion in the left adnexa with enhancing solid components (arrow).

**Figure 2:**
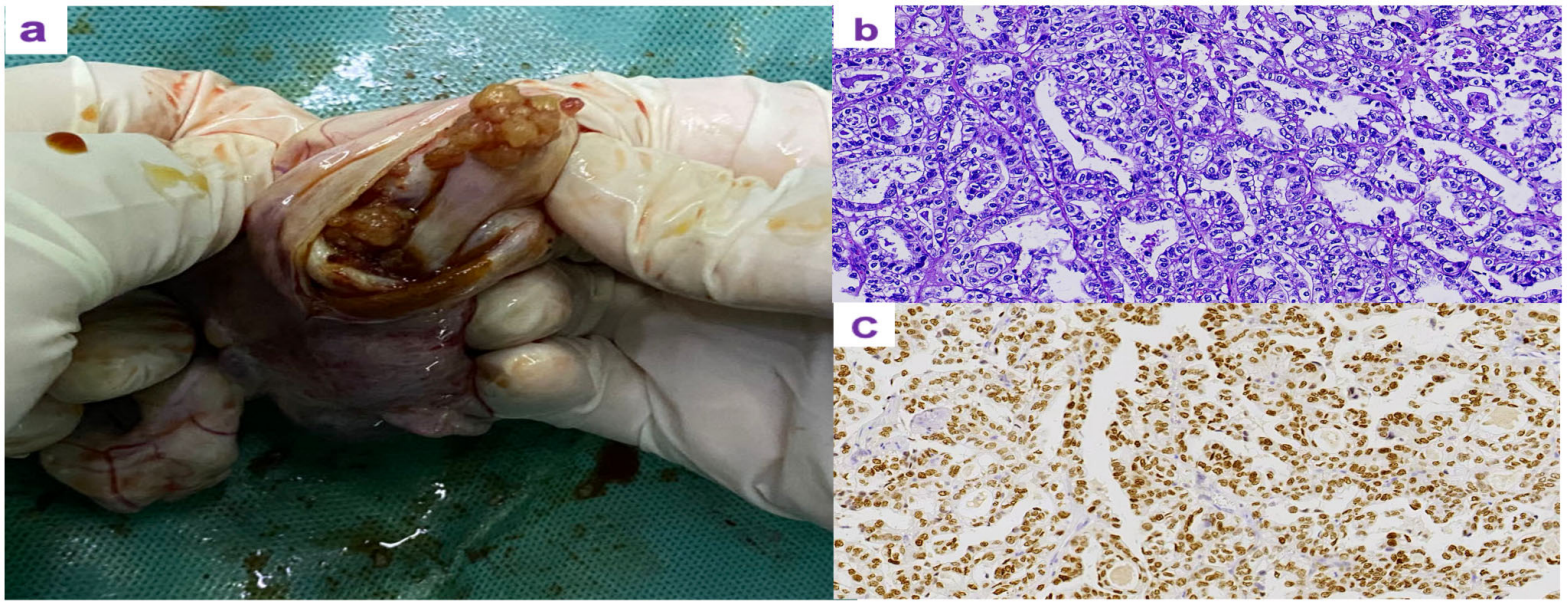
Pathological images of the tumor. **(a)** Gross appearance of the opened tumor. **(b)** Hematoxylin and eosin (HE) staining showing the tumor composed of polygonal and hobnail-shaped cells with clear cytoplasm arranged in tubular, papillary, and solid patterns. **(c)** Immunohistochemical staining for HNF-1*β* showing diffuse positive nuclear expression.

A 33-year-old female patient, unmarried, G0P0 came to the gynecological department with the main complaint of left adnexal mass discovered on physical examination 7 days ago. She complained occasional mild lower-abdominal pain between menstruations, without bloating, weight loss, and abnormal vaginal bleeding. The past history did not show abnormalities. Her family history included her father suffering from hypertension and coronary heart disease, while her mother was healthy. At her first visit, transvaginal ultrasonography revealed a 7.5 × 4.4 cm mixed cystic–solid mass in the left adnexal region with internal septations and increased vascularity on color Doppler imaging (**Figure 1a**). Pelvic magnetic resonance imaging (MRI) further confirmed a cystic–solid lesion in the left adnexa (**Figure 1b**). The laboratory results showed that CA-125, CA-153, CA-199, HE4, CEA, and AFP were all in normal range. Because of the patient’s young age and early-stage disease, a laparotomic left salpingo-oophorectomy was performed to minimize the risk of intraoperative tumor rupture and to allow complete oncologic staging in the setting of a suspected malignant ovarian mass. Approximately 50 mL of brown ascitic fluid was observed intraoperatively and sent for cytological examination, which was negative for malignant cells. No macroscopic peritoneal implants or nodules were identified during systematic exploration of the peritoneal cavity. The ovarian mass was removed intact without rupture and then opened after excision. Gross examination revealed a solid–cystic mass with a cystic wall encapsulating solid components (**Figure 2a**). Histopathological examination of hematoxylin and eosin-stained sections showed tumor cells with clear cytoplasm and hobnail morphology, arranged in tubular, papillary, and solid patterns, consistent with OCCC (**Figure 2b**). Immunohistochemical staining demonstrated strong diffuse nuclear positivity for HNF-1*β* (**Figure 2c**). The comprehensive surgical staging was immediately performed after intraoperative pathological confirmation by frozen section, including systematic pelvic and para-aortic lymphadenectomy involving bilateral pelvic, bilateral para-aortic, and bilateral common iliac lymph nodes. A total of 49 lymph nodes were examined histopathologically, all of which were negative for metastasis. In addition, a complete omentectomy was performed, and pathological examination of the omentum revealed no evidence of malignancy. The final histopathological examination confirmed the diagnosis of OCCC, FIGO stage IA (pT1a N0 M0).

**Figure 3:**
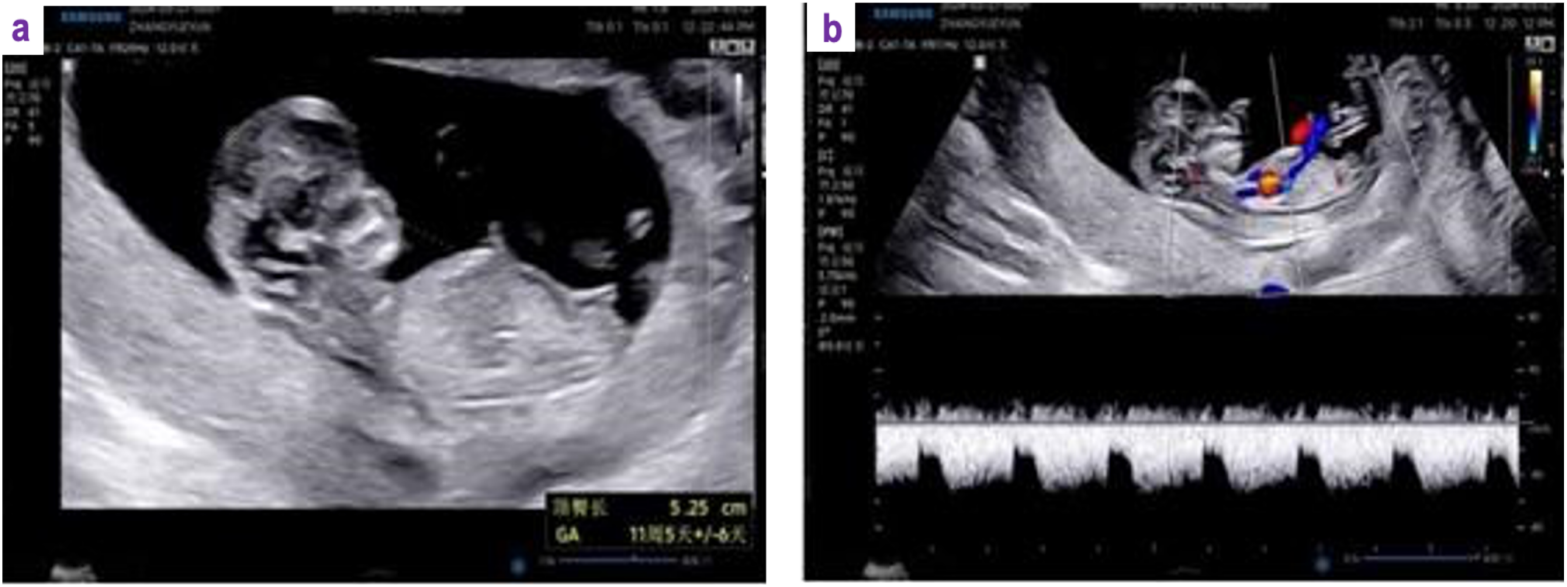
Ultrasound examination confirming successful pregnancy after fertility-sparing surgery. **(a)** A viable intrauterine pregnancy observed at 11$^{+}$4 weeks of gestation, with a crown–rump length of 52 mm and normal fetal cardiac activity. **(b)** Transvaginal ultrasound showing a cystic lesion in the right ovary measuring approximately 48 × 31 mm with homogeneous internal echoes.

Following surgery, the patient received six cycles of adjuvant chemotherapy consisting of liposomal paclitaxel (175 mg/m^2^ intravenously on Day 1) and carboplatin (AUC 6 intravenously on Day 2) administered every 3 weeks. Chemotherapy was completed without significant complications. To preserve ovarian function during chemotherapy, a gonadotropin-releasing hormone agonist, dGnRH-*α* (leuprolide acetate, 3.75 mg), was initiated 2 weeks after the first cycle of chemotherapy and administered subcutaneously every 28 days for a total of six doses. After completion of chemotherapy, the patient was followed with regular surveillance, including physical examination, pelvic ultrasonography, and serum tumor markers (CA-125 and CA19-9) every 3 months. No evidence of disease recurrence was detected during follow-up. The patient subsequently underwent *in vitro* fertilization and embryo transfer (IVF-ET) at our institution’s reproductive medicine center. In April 2024, the patient was counseled to delay conception for 12 months after completion of chemotherapy, and a viable intrauterine pregnancy was confirmed by ultrasound following the second embryo transfer (**Figure 3**). The pregnancy resulted in a full-term delivery, and the patient gave birth by cesarean section without complications. A comparison of the present case with previously published fertility-sparing OCCC cases is summarized in **Table 1**.

**Table 1:** Comparison of the present case with previously published fertility-sparing ovarian clear cell carcinoma case reports.

	Present case	Chang et al., (11)	Nishida et al., (12)
FIGO stage	IA (PT1a, N0, M0)	IA (PT1a, N0, M0)	IA (PT1a, N0, M0)
Pathological type	OCCC	OCCC	OCCC
Tumor size (mm)	72	60	80
Tumor laterality	Unilateral	Unilateral	Unilateral
LN dissection/number (*n*)	Yes (32)	Yes (NR)	Yes (91)
Omentectomy type	Complete	Infracolic	Partial
Peritoneal cytology	Negative	Negative	Negative
Chemotherapy regimen/cycles	TC ×6	TC ×6	TC ×4
GnRH agonist use	Yes	No	No
Pregnancy outcome	Pregnancy	No	No
Pregnancy method	Spontaneous live birth	–	–
Follow-up duration (months)	36	24	108
Recurrence or *de novo* development time after surgery (months)	No recurrence	24	108
Recurrence site	–	Ipsilateral pelvic region	Contralateral ovary

NR, not reported; LN, lymph node; TC, paclitaxel (175 mg/m^2^)/carboplatin (AUC 6); GnRH, gonadotropin-releasing hormone.

## 3 Discussion

In this case report, we described a rare but encouraging case of successful pregnancy following FSS and adjuvant chemotherapy in a patient with FIGO stage IA OCCC. This outcome is clinically meaningful given the traditionally aggressive behavior of OCCC and the limited evidence supporting fertility preservation in this histologic subtype. Although FIGO stage IA OCCC is associated with a more favorable prognosis compared with advanced-stage disease, recurrence remains a clinically relevant concern. Reported recurrence rates for stage IA OCCC range from approximately 5% to 15% in retrospective series, with most recurrences occurring within the first 2–3 years after initial treatment. Factors consistently associated with increased recurrence risk include higher tumor stage, intraoperative capsule rupture, incomplete surgical staging, residual disease, and unfavorable molecular features such as PIK3CA mutations and aberrant HNF-1*β* expression. These findings underscore the importance of meticulous surgical technique, comprehensive staging, and careful patient selection when considering fertility-sparing approaches in early-stage OCCC. Although OCCC has historically been considered a high-risk subtype, emerging retrospective data suggest that FSS may be oncologically safe in carefully selected young patients with stage I disease who wish to preserve fertility. Comparative studies have shown no significant differences in recurrence patterns, disease-free survival, or overall survival between patients undergoing FSS and those treated with radical surgery, particularly in early-stage OCCC (10, 13). More recently, Manning-Geist et al. analyzed oncologic outcomes of FSS in patients with early-stage OCCC using a large population-based database. Their study suggested that, although FSS may be feasible in carefully selected patients with stage IA disease, an increased risk of recurrence could not be definitively excluded, particularly in incompletely staged cases. Importantly, the authors emphasized that tumor stage, surgical completeness, and patient selection are critical determinants of oncologic safety, and advised caution when considering fertility-sparing approaches in OCCC. These findings align with the present case, in which comprehensive surgical staging and the absence of high-risk features may have contributed to the favorable oncologic outcome. While **Table 1** focuses on individual case reports with detailed reproductive outcomes, larger cohort studies provide complementary evidence regarding oncologic safety and fertility outcomes after FSS in OCCC. Manning-Geist et al. (14) reported population-level data, suggesting that FSS may be feasible in carefully selected patients with stage IA disease, although an increased recurrence risk could not be excluded. In addition, Wang et al. (15) reported reproductive outcomes in a cohort of patients undergoing fertility-sparing management, in which six patients attempted conception and two (33.3%) achieved term pregnancies (**Table 2**). Together, these cohort data support the feasibility of fertility preservation in selected patients while underscoring the importance of strict patient selection and comprehensive staging.

**Table 2:** Selected cohort studies evaluating fertility-sparing surgery and reproductive outcomes in OCCC.

Study	Manning-Geist et al. (14)	Wang et al. (15)
Design	Population-based cohort	Retrospective cohort
OCCC patients (*n*)	182	55
Stage	IA–IC	IA–IC
Attempted conception (*n*)	NR	6
Successful pregnancies (*n*, %)	NR	2 (33.3%)
Key oncologic finding	FSS feasible in selected stage IA; caution advised	No compromise in short-term oncologic safety

NR, not reported.

In addition, aberrant p53 expression has been reported as a potential marker of aggressive behavior and increased recurrence risk in OCCC. However, no high-risk p53-associated features were identified in the present case. These findings support the notion that tumor stage and completeness of surgical staging may be more critical determinants of prognosis than the extent of ovarian resection alone. In the present case, comprehensive staging, including extensive lymphadenectomy, omentectomy, and peritoneal cytology, likely contributed to both accurate risk stratification and favorable oncologic outcome.

Despite early-stage diagnosis, OCCC remains characterized by intrinsic resistance to platinum-based chemotherapy, with reported response rates as low as 6%–11% in advanced or recurrent settings and poor survival outcomes in platinum-resistant disease (16). Retrospective data indicate that the median overall survival of patients with platinum-sensitive OCCC is approximately 16 months, whereas that of patients with platinum-resistant disease is only 7 months (9). Together, these findings underscore the need for novel targeted therapies to improve outcomes in OCCC. Current international guidelines generally discourage FSS in OCCC due to its aggressive biological behavior and relative chemoresistance. The NCCN, ESM-ESGO, FIGO, and Japanese guidelines recommend FSS primarily for patients with stage IA, grade 1–2 EOCs of endometrioid or mucinous histology, and consider OCCC a high-risk subtype in which FSS should be avoided except in highly selected cases. In the present case, FSS was undertaken in the setting of FIGO stage IA disease with comprehensive surgical staging and after detailed patient counseling. This case does not challenge existing guidelines but highlights the need for individualized treatment regimen in rare and carefully selected circumstances.

Yin et al. published in 2019 that pregnancy after FSS has been encouraged for patients with stage IA/IC low-grade EOC. In one cohort of 40 patients (mean age, 28 years; range, 18–44 years), histologic subtypes included mucinous (47.5%), clear cell (22.5%), endometrioid (20%), and serous carcinoma (10%). Laparoscopic surgery was performed in 40% of cases, and 67.5% of patients received adjuvant chemotherapy. Among those who attempted conception (13 patients, 32.5%), eight (61.5%) achieved spontaneous pregnancy, resulting in five live births (three term and two preterm), one fetal loss, one intrauterine fetal death, and one fetal anomaly. Notably, no tumor recurrence occurred during pregnancy (17).

In this case, the patient received six cycles of paclitaxel (175 mg/m²) and carboplatin (AUC 6) as adjuvant chemotherapy, which were completed without complications. Three months after completion of treatment, all laboratory parameters had returned to normal. Concurrently, a gonadotropin-releasing hormone agonist (GnRH-*α*; leuprolide depot) was administered every 28 days for a total of six doses to protect ovarian function. Although the benefit of adjuvant chemotherapy for stage IA–IC1 OCCC remains uncertain because of the relative chemoresistance of this subtype compared with other forms of EOC, chemotherapy was administered in this case in view of the potential risk of microscopic residual disease and the inherently aggressive biological behavior of OCCC. Although several case reports and retrospective series have described FSS in patients with early-stage OCCC, such reports remain limited and heterogeneous in terms of staging completeness, adjuvant treatment, and reproductive outcomes. The present case adds to the existing literature by providing a comprehensively staged FIGO stage IA OCCC, including extensive pelvic and para-aortic lymphadenectomy, complete omentectomy, and negative peritoneal cytology, followed by adjuvant platinum-based chemotherapy with ovarian protection and successful post-treatment pregnancy. This case highlights the feasibility of an integrated multidisciplinary approach and contributes additional evidence supporting the oncologic safety and reproductive potential of fertility-sparing management in carefully selected patients with early-stage OCCC.

## 4 Conclusion

This case describes a rare favorable reproductive outcome following fertility-sparing management in early-stage OCCC. Given the aggressive biology of OCCC and limited supporting evidence, such outcomes should not be generalized. FSS should be reserved for highly selected patients after comprehensive staging, thorough counseling, and shared decision-making, with management in experienced centers and long-term surveillance.

## Data Availability

The original contributions presented in the study are included in the article/supplementary material. Further inquiries can be directed to the corresponding authors.
